# Body Size Evolution in Extant Oryzomyini Rodents: Cope's Rule or Miniaturization?

**DOI:** 10.1371/journal.pone.0034654

**Published:** 2012-04-03

**Authors:** Jorge Avaria-Llautureo, Cristián E. Hernández, Dusan Boric-Bargetto, Cristian B. Canales-Aguirre, Bryan Morales-Pallero, Enrique Rodríguez-Serrano

**Affiliations:** Laboratorio de Ecología Molecular & Filoinformática, Departamento de Zoología, Facultad de Ciencias Naturales y Oceanográficas, Universidad de Concepción, Concepción, Chile; University of Lausanne, Switzerland

## Abstract

At the macroevolutionary level, one of the first and most important hypotheses that proposes an evolutionary tendency in the evolution of body sizes is “Cope's rule". This rule has considerable empirical support in the fossil record and predicts that the size of species within a lineage increases over evolutionary time. Nevertheless, there is also a large amount of evidence indicating the opposite pattern of miniaturization over evolutionary time. A recent analysis using a single phylogenetic tree approach and a Bayesian based model of evolution found no evidence for Cope's rule in extant mammal species. Here we utilize a likelihood-based phylogenetic method, to test the evolutionary trend in body size, which considers phylogenetic uncertainty, to discern between Cope's rule and miniaturization, using extant Oryzomyini rodents as a study model. We evaluated body size trends using two principal predictions: (a) phylogenetically related species are more similar in their body size, than expected by chance; (b) body size increased (Cope's rule)/decreased (miniaturization) over time. Consequently the distribution of forces and/or constraints that affect the tendency are homogenous and generate this directional process from a small/large sized ancestor. Results showed that body size in the Oryzomyini tribe evolved according to phylogenetic relationships, with a positive trend, from a small sized ancestor. Our results support that the high diversity and specialization currently observed in the Oryzomyini tribe is a consequence of the evolutionary trend of increased body size, following and supporting Cope's rule.

## Introduction

Body size is one of the most significant traits of animals, not only because it is correlated with many life-history and ecological characteristics [1]–[4], but also given its importance in the evolution of taxa [5], [6]. At the macroevolutionary level, one of the first and most important hypotheses that proposes an evolutionary tendency in the evolution of body sizes is “Cope's rule" [3], [7]. This rule predicts that the size of species within a lineage increases over evolutionary time [8]–[13]. In fact, using fossil data from Cenozoic North American mammals, Alroy [9] found that on average, the increment of body size from ancestor to descendant is 9%. Explanations for Cope's rule principally include: 1.- the consideration that the greatest body sizes have the greatest fitness (*e.g.*, [14]); and 2.- an increase in the variance of body size is the result of diversification by passive diffusion from a small sized ancestor (*e.g.*, [8], [15], [16]). The first explanation generates the prediction that the distribution of forces and/or constraints that affect the tendency are homogenous [16] and generate a directional process [17]. The second explanation predicts that there is no dominant force determining the evolutionary tendency. In this sense, the distribution of forces and/or restrictions that affect the tendency would be heterogeneous [16] and generate a random process [17] in which the only restrictions are given by the minimum viable body size and the small sized common ancestor [8], [15], [16], [18].

Alternatively, an important, but less explored, pattern of body size evolution is miniaturization [5], for which there is ample evidence in a variety of taxa [19]–[22], for a review see 5]. This trend has been understood as an extreme reduction of body size, leading to a shift in physiology, ecology, life history and behavioral traits. However, a less severe reduction in body size can also be part of a miniaturization trend, which can be assessed in an explicit phylogenetic framework [5], [23], [24]. The consequences of miniaturization are results of heterochronic underlying process that induce bauplan reduction or simplification, phenotypic novelties, as well as the increase in variability of late ontogeny elements that can facilitate diversification [5]. These alternative historical body size trends (*i.e.*, Cope's rule versus miniaturization) suggest that in a monophyletic group the processes that generate these patterns are based on selection or any other within-lineage process that has a directional tendency [25].

Both Cope's rule and miniaturization have considerable empirical support in the animal fossil record [9], [11]–[13], [23], [26]–[28]. However, Monroe & Bokma [29] find no evidence for Cope's rule in extant mammals using a single phylogenetic tree approach and a Bayesian based model of evolution. Their study analyzed the evolution of body size in 4,554 existing mammal species, in a phylogenetic tree that comprises nearly all mammals, and is currently the most comprehensive of its kind using extant species. This is interesting given that their results contrast with the fossil evidence of mammals found by Alroy [9], Smith *et al.* [28] and McFadden [30].

This contrasting result could be due to the use of a one phylogenetic tree approach, which implies that parameters that define the evolutionary patterns were estimated assuming that the phylogeny or the evolutionary history of the group under study is known without error [31], [32]. Nevertheless, phylogenies are rarely known with complete certainty [33], and are usually inferred from groups of morphological or molecular data [34], which are themselves subject to error and uncertainty. This presents a problem because different phylogenetic trees could give different answers to the same comparative questions. As a result, all of the conclusions derived from the comparative analyses using a single phylogenetic tree are conditional to this particular phylogeny. It has recently been suggested that the Bayesian method using Markov Chain Monte Carlo (hereafter BMCMC) offers a solution to the problem of sampling phylogenies by using a formal statistical procedure to sample from the probability of phylogenetic trees [31], [35]–[39]. This method can be applied to comparative analyses for the study of ancestral character reconstruction [40]–[44], and potentially to evaluate modes and patterns of character evolution, such as evolutionary trends in body size.

Here we utilize the phylogenetic comparative method, incorporating phylogenetic uncertainty, to assess Cope's rule/miniaturization using extant Sigmodontinae (Rodentia: Cricetidae) rodents of the Oryzomyini tribe as a study model. Oryzomyini are the most diverse tribe of endemic rodents in South America, with about 121 species and 28 genera [45]. It is also the most widely distributed tribe, from Tierra del Fuego to the southern United States, including many oceanic islands [46], [47]. This group inhabits a variety of environments, including tropical and temperate rain forests, subtropical and desert open areas and the high Andean plateau. Species have a wide range of body sizes, ranging from 62 to 254 mm [47]. To date there has only been one empirical evaluation with respect to body size evolution in this monophyletic group, which suggested that current body size diversity originated from a medium sized ancestor [47]. This neontological study was based on a single phylogenetic tree approach and parsimony to reconstruct ancestral nodes. Nevertheless, such reconstruction techniques have been shown to be inadequate in the study of evolutionary trends, as they are constrained to reconstruct ancestral nodes at values that are intermediate to values in extant species [48]–[52], and assume a simple random walk model. However, evaluating whether the diversification of the traits are the result of random or directional (*i.e.*, an increase or decrease) diversification mechanisms, has important implications for understanding the origin and diversification of lineages [16], [52]–[57]. To this respect, a new method proposed by FitzJohn [25] based on the birth-death processes, allows for evaluation of Cope's rule/miniaturization hypothesis, and their relationship with diversification rate.

We utilized this recently developed phylogenetic birth-death method (quantitative state speciation and extinction [QuaSSE]) proposed by FitzJohn [25], to test the evolutionary trend in body size, incorporating the uncertainty of phylogenetic trees obtained from Bayesian analysis, and to compare with a single phylogenetic tree approach, by assessing body size evolution in a Maximum Likelihood and Bayesian consensus tree. We evaluated Cope's rule/miniaturization using two principal predictions: (a) phylogenetically related species are more similar in their body size, than expected by chance; (b) body size increased/decreased over time. Consequently the distribution of forces and/or constraints that affect the tendency are homogenous and generate this directional process from a small/large sized ancestor.

## Materials and Methods

### Body size and DNA data collection

We compiled a bibliographic database containing the maximum head-body length for 51 extant Oryzomyini species for which molecular data was available for phylogenetic analyses (Table S1). We selected this trait because it is less temporally variable than other metrics such as body mass [52], and because it is robustly related to overall body size distribution, and hence to mean and median body size [58]. Analyses were performed using natural logarithm transformation of the data in millimeters to normalize.

We used sequences of the Interphotoreceptor retinoid-binding protein nuclear gene, and Cytochrome-*b* mitochondrial gene (hereafter IRBP, and Cyt *b*, respectively) from GenBank [59], because sequences are available for a large number of Oryzomyini species (Table S1). Four sigmodontines pertaining to other tribes (*Calomys callosus*, *Bibimys labiosus*, *Juliomys pictipes*, and *Eligmodontia typus*) were selected as outgroups, based on the phylogeny of D'Elia [60], D'Elia *et al.* [61], and Smith & Patton [62] (Table S1).

### BMCMC molecular phylogeny and estimates of divergence times

Given that Oryzomyini diversification has been dated around 7 million years ago [63], [64], the selected molecular marker could be saturated, and provide spurious phylogeny. Therefore we evaluated whether the sequences were saturated and thus useful for the phylogenetic analysis, using Xia's test [65] implemented in DAMBE v5.1.5 [66]. This is an entropy-based index that estimates a substitution saturation index (Iss) and compares it to a critical substitution saturation index (Iss.c) via a randomization process with 95% confidence intervals [67], [68]. We used concatenated aligned DNA sequence data of the IRBP-Cyt *b* genes from the 55 species, corresponding to the ingroup and outgroups (Table S1). We aligned the sequences using Clustal W [69], and by eye. Additionally, given that this study did not contain all known current species, we performed the node-density artifact test to determine any possible effect caused by missing taxa on the reconstructed sample of phylogenetic trees [70]. This test fits two parameters that describe the rate of change between the path lengths of a tree and the number of nodes (β*), and the curvature of this relationship (δ). When the data do not suffer from sparse taxon sampling the parameters should be β*>0 and δ≤1 [71].

With concatenated aligned sequences we simultaneously estimated phylogenetic relationships, branch lengths, and divergence times for the Oryzomyini tribe using BEAST 1.6.2 software [72]. This analysis was conducted using a BMCMC framework to estimate the posterior probability of phylogenetics trees, and includes this uncertainty in the comparative analysis. As prior information we used a GTR+Γ+I model of sequence evolution, the Yule process of speciation, and two points of fossil calibration: 1.6±0.64 Mya. for the origin of *Oligoryzomys flavescens* (see [73]), and 1.5±0.8 Mya. for the origin of *Holochilus* (see [74]). Analyses were based on four models of mutation rate: 1) A strict molecular clock; 2) an uncorrelated lognormal relaxed clock; 3) an uncorrelated exponential relaxed clock; and 4) a random local clock. The MCMC chain was run for 21,000,000 generations (10,000 generations were discarded as burn in, before the posterior probabilities distribution of the selected diversification model converged), with parameters sampled every 10,000 steps, and we resampled every 15 trees to obtain a final sample of 139 trees, with an autocorrelation of 0.006 in ln-likelihood of the sample. Examination of MCMC samples using TRACER v. 1.5 software [75] suggested that the independent chains were each adequately sampling the same probability distribution; and that effective sample sizes for all parameters of interest were greater than 500. In order to find the best molecular clock model we used Bayes factor [76] to compare the four clock models, given that it is the soundest theoretical framework for model comparison in a Bayesian framework [72].

### Evaluation of evolutionary trends

Using the GLS model [49], [77]–[80] we first evaluated the influence of phylogeny or phylogenetic signal on body size with the phylogeny scaling parameter λ [49], [79], [81], using the consensus tree obtained from Bayesian analyses. The parameter λ evaluates the extent to which the phylogeny correctly predicts the patterns of similarity of traits among species [49], [79], or if the traits are evolving according to the PGLS model on the given phylogeny [81]. In this approach λ reveals whether the phylogeny fits to the patterns of covariance among close species for a given trait. This analysis is based on the V matrix of variance–covariance, where the variance is assumed to be directly proportional to the sum of the branch lengths from the root to the tips. The expected covariance between any two species is assumed to be proportional to the sum of their shared branch lengths, and the parameter λ is the multiplier of the elements outside the diagonal of V. This parameter evaluates one of the key assumptions underlying the use of the Comparative Method (*i.e.*, that species are not independent). If trait evolution is independent of phylogenetic relationships, then this parameter will take the value of 0, indicating that a trait's values are not predicted by the ancestor-descendant relationships and, consequently, analysis of body size using the phylogeny is not appropriate [80]. If traits are evolving as expected, given the tree topology and branch length, λ takes the value of 1 [81].Values of λ between 0 and 1 indicate different levels of phylogenetic signal [49]. The random-walk model with the observed λ parameter was contrasted with the random-walk model forcing λ = 0; and λ = 1, respectively.

Using the birth-death model [25] implemented in a maximum likelihood framework we evaluated body size evolution and its relationship with speciation rate over the Bayesian sample of ultrametric-phylogenetic trees. This model takes a phylogeny and set of trait measurements for the tip species, and fits a series of birth–death models in which the speciation and extinction probabilities are independent of trait evolution, or vary along branches as a function of a continuous trait that evolves according to a diffusion process, with or without an evolutionary tendency (*i.e.*, increase or decrease over time) [25]. The anagenetic component of character evolution is incorporated by the change described in the diffusion model, and the cladogenetic component is incorporated with the effect of speciation event in the estimation of ancestral character states. To test if a directional tendency in body size evolution exists we compared models in which speciation rates were constant and independent of body size evolution, and where speciation rates vary as a linear, sigmoidal, or hump-shaped function of body size, evolving by a diffusion process, with and without a directional trend. In a linear model the speciation rate varies proportional to body size, in a sigmoidal model smaller species have a low speciation rate compared to larger species, and in a hump model species with the mean body size have the highest speciation rate.

Specifically we used seven models of speciation rate: 1) a constant model of speciation; 2) speciation that varies as a linear function of body size, evolving by a diffusion process; 3) speciation that varies as a sigmoidal function of body size, evolving by a diffusion process; 4) speciation that varies as a hump function of body size, evolving by a diffusion process; 5) speciation that varies as a linear function of body size, evolving by a diffusion process with a directional trend; 6) speciation that varies as a sigmoidal function of body size, evolving by a diffusion process with a directional trend; and 7) speciation that varies as a hump function of body size, evolving by a diffusion process with a directional trend. These models have the following parameters: the speciation and extinction rate parameters (λ_S_, μ); the diffusion parameter (σ^2^), which is the expected squared rate of change and captures the stochastic elements of character evolution; and the directional trend “drift" parameter (θ), which captures the deterministic or directional component of character evolution, this is the expected rate of change of the character over time and may be due to selection or any other within-lineage process that has a directional tendency [25]. These models were implemented in QuaSSE software [25], and the analyses were done using the Diversitree package of R software [82]. The parameter values of birth-death models were estimated on each tree of the sample of trees (Nexus trees S1) from the BMCMC molecular phylogenetic approach implemented in BEAST (*i.e.*, the analyses were done 139 times). To select the best model that describes body size evolution in the sample of trees we used Bayes Factor. Given issues with the power for detecting and estimating extinction from molecular phylogenies [25], [83], we did not test extinction-variable models.

Finally, to compare the obtained results based on a sample of trees with a single phylogenetic tree approach, we assessed body size evolution in a Maximum Likelihood (ML), and Bayesian consensus (BC) tree. For these one phylogenetic tree approaches we selected the best model using the Akaike information criterion (AIC).

## Results

**Figure 1:**
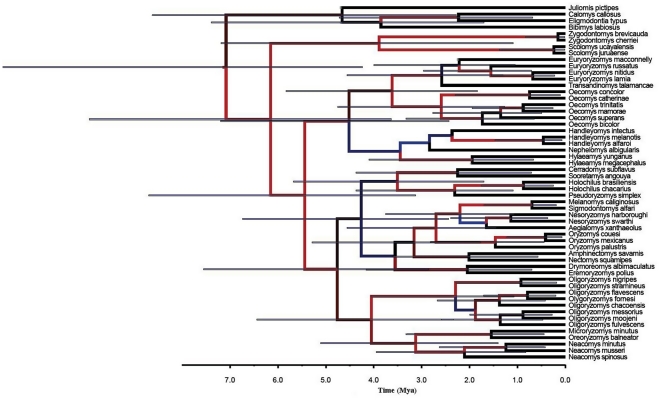
Bayesian consensus tree obtained from 139 ultrametric trees based on an uncorrelated exponential relaxed clock. Blue branches indicate posterior probability values of a node below 0.5. Horizontal blue bars indicate the 95% HPD of divergence times, and the scale axis shows divergence times as millions of years ago (Mya).

The analyzed IRBP-Cyt *b* sequences presented low saturation, as the critical index of substitution saturation values (Iss.c = 0.469) was significantly higher than the observed index of substitution saturation values (Iss = 0. 209; p<0.0001), therefore, the sequences are suitable for performing phylogenetic analyses. The Bayes factor analysis suggests that, for this data set, the relaxed molecular clock model using exponential distribution is both a more precise estimator and has a better fit to the data (Table 1). The consensus tree obtained from this clock model (Fig. 1), showed high posterior probability for the majority of nodes, and the topology and divergence time estimates were consistent with previous work [63], [64]. Maximum likelihood, Bayesian consensus and the sample of trees are available as a Nexus file in Nexus Trees S1. The node-density artifact analysis did not detect evidence of an effect caused by missing taxa or sparse taxon sampling in the reconstructed phylogenies (δ<1 when β is significant = 25.15%).

**Table 1:** Bayes Factors used to test the four molecular clock models.

	Strict	Exponential	Lognormal	Local Clock
Strict	-	0.0	0.0	0.1
**Exponential**	**48494480.2**	-	**10454.724**	**5471192.3**
Lognormal	4638.5	0.0	-	523.3
Local Clock	9.0	0.0	0.0	-

Values≥3 give support for the model listed in the first column, values≤−3 give support for the row model. Strict = Strict clock model; Exponential = Uncorrelated exponential relaxed clock; Lognormal = Uncorrelated lognormal relaxed clock; and Local clock = Random local clock model.

**Figure 2:**
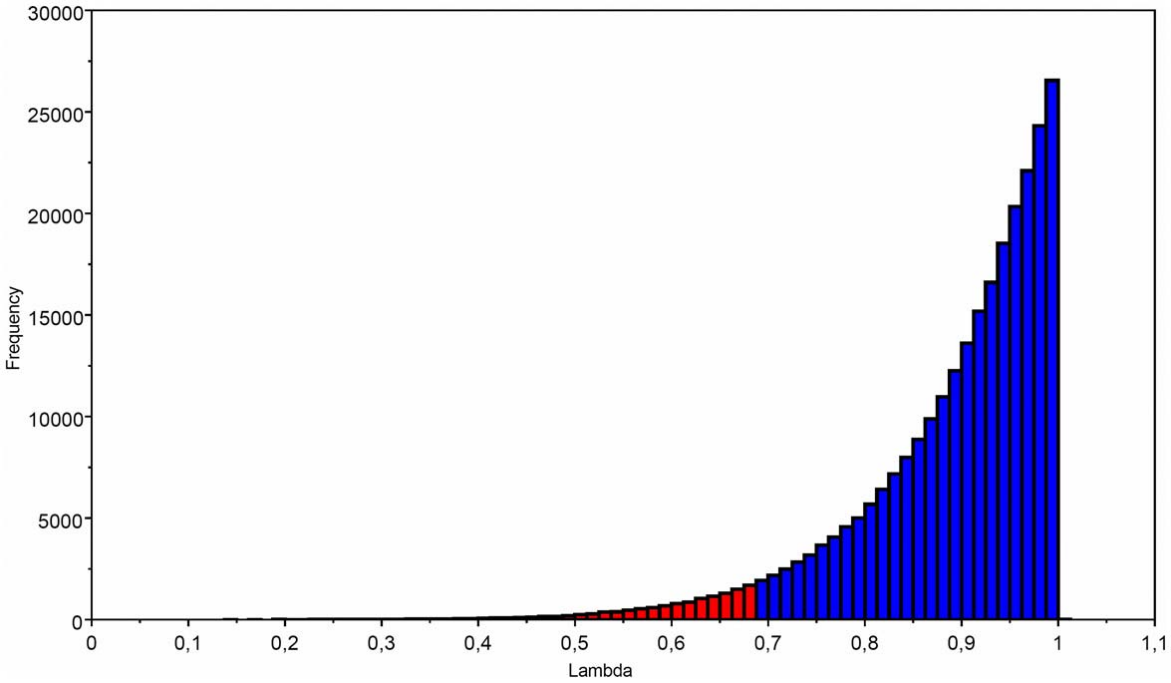
Bayesian posterior probability distribution for the lambda (λ) parameter based on the ultrametric Bayesian consensus tree. Vertical blue bars indicate the 95% HPD.

The variability of body size was significantly influenced by phylogenetic relationships (λ = 1; Table 2; Fig. 2). The Bayes Factor comparison between the seven birth-death models, that considered uncertainty in phylogenetic trees, showed that the best predictor of body size evolution in Oryzomyini rodents was a Drift Linear model with a positive trend (θ = 0.33; Table 3, 4), where larger species have higher speciation rates. Similarly, analyses based on a single phylogenetic tree approach and AIC values (Table 5, 6), showed that the same Drift Linear model with a positive trend was the best predictor of body size evolution (AIC = 135.1 and 140.5 for Bayesian consensus and Maximum likelihood trees, respectively). The positive trend for body size evolution was described by θ = 0.48, and θ = 0.45 for Bayesian consensus and Maximum likelihood trees, respectively (Table 5, 6).These results support the general tendency to increase body size over time, and that larger species have a higher speciation rate.

**Table 2:** Bayes Factors used to test the observed versus expected values of the phylogenetic scaling parameter λ based on Random Walk model.

	Ln Harmonic mean	Bayes Factor
Observed λ = 0.89 (0.68; 1)	7.4	-
Forced λ = 0	−4.9	24.6
Forced λ = 1	9.3	−3.8

The observed λ (mean; 95% HPD) were contrasted with values expected under the hypotheses of no phylogenetic signal (λ = 0) and the pure Random Walk model (λ = 1).

Bayes Factor (BF)≥3 indicates support for the observed λ parameter. When BF is ≤−3 the other model is chosen. Observed λ was contrasted versus λ = 0, and λ = 1.

**Table 3:** Bayes Factors used to test the speciation rate models taking into account uncertainty of phylogenetic trees.

	Full	Linear	Sigmoidal	Hump	Drift Linear	Drift Sigmoidal	Drift Hump
Full	-	0.7	0.7	0.3	0.0	0.0	0.0
Linear	1.4	-	1.0	0.4	0.0	0.0	0.0
Sigmoidal	1.4	1.0	-	0.3	0.0	0.0	0.0
Hump	3.9	2.9	2.9	-	0.0	0.1	0.1
**Drift Linear**	**817.1**	**599.6**	**602.1**	**209.7**	**-**	**22.4**	**14.4**
Drift Sigmoidal	36.5	26.8	26.9	9.4	0.0	-	0.6
Drift Hump	56.8	41.7	41.9	14.6	0.1	1.6	-

Values≥3 give support for the first model listed in the column, values≤−3 give support for the row model. Full = constant speciation rate; Linear, Sigmoidal, Hump = speciation varies as a function of body size, evolving by a diffusion process, with a linear, sigmoidal or hump function, respectively; Drift Linear, Drift Sigmoidal, Drift Hump = speciation varies as a function of body size, evolving by a diffusion process with a directional trend (Drift).

**Table 4:** Mean Drift parameter observed for three speciation rate models.

	Drift Linear (θ)	Drift Sigmoidal (θ)	Drift Hump (θ)
Mean Drift	0.33	0.34	0.36
95% HPD	−0.6; 0.7	−0.5; 0.9	−0.03; 0.8

Parameters were estimated using a maximum likelihood approach in each tree of the Bayesian sample.

**Table 5:** Maximum likelihood parameter estimation and Akaike information criterion (AIC) values used to select the best model of speciation rate based on the Bayesian consensus tree.

	Df	Ln Lik	AIC	ChiSq	Drift (θ)	Pr(>[Chi])
Full	3	−70.34	146.68	-	-	-
Linear	4	−70.11	148.23	0.45	-	0.503
Sigmoidal	6	−70.10	152.2	0.47	-	0.925
Hump	6	−69.38	150.75	1.92	-	0.589
**Drift Linear**	5	−62.56	**135.12**	15.56	0.48	**0.000**
Drift Sigmoidal	7	−64.28	142.56	12.11	0.48	**0.017**
Drift Hump	7	−61.80	137.59	17.08	0.48	**0.002**

Df = Degrees of freedom of each model; lnLik = Natural logarithm of Maximum Likelihood; AIC = Akaike information criterion; ChiSq = Chi Square value; Drift = tendency of body size evolution; and Pr(>[Chi]) = Chi-square probability value.

**Table 6:** Maximum likelihood parameter estimation and Akaike information criterion (AIC) values used to select the best model of speciation rate based on the Maximum likelihood tree.

	Df	Ln Lik	AIC	ChiSq	Drift (θ)	Pr(>[Chi])
Full	3	−71.78	149.55	-	-	-
Linear	4	−71.49	150.98	0.57	-	0.451
Sigmoidal	6	−71.49	154.99	0.56	-	0.906
Hump	6	−70.77	153.54	2.01	-	0.570
**Drift Linear**	5	−65.28	**140.56**	12.99	0.45	**0.001**
Drift Sigmoidal	7	−66.57	147.13	10.42	0.45	**0.034**
Drift Hump	7	−65.10	144.20	13.35	0.45	**0.009**

Df = Degrees of freedom of each model; lnLik = Natural logarithm of Maximum likelihood; AIC = Akaike information criterion; ChiSq = Chi Square value; Drift = tendency of body size evolution; and Pr(>[Chi]) = Chi-square probability value.

## Discussion

There has been a succession of improving studies regarding the evolutionary processes that gave rise to the current patterns of biodiversity in Oryzomyini rodents, which has primarily focused on the time of origin, biogeography and phylogenetic affinities of several species [47], [60]–[62], [85], [86]. Some progress has been made towards understanding these processes, especially through the discovery of fossil records [74], [84], [85], [87]–[95]. However, the incomplete fossil record of early Oryzomyini forms has hindered the description of the evolutionary history of this tribe. This difficulty is augmented when researchers attempt to understand the evolutionary history of particular traits like body size, since there are no fossil data that allow researchers to evaluate ancient character evolution and diversification [70]. Our results –based on extant species and the use of the comparative method in a sample of Bayesian trees– suggest that the current body size distribution in the oryzomyine tribe is explained by phylogenetic relationships (*i.e.*, phylogenetic signal, Table 2; Fig. 2), and that during the evolutionary history of oryzomyines there was an evolutionary trend towards increased body size, which suggests that the distribution of forces and/or constraints that determine the tendency for larger body size over time are homogenous and generate a directional process (*i.e.*, Cope's rule hypothesis, Table 3, 4). Alternatively, it is also possible that the increase in the body size of oryzomine rodents is caused by passive diffusion from the minimum viable body size, as postulated by Stanley [8] (but see [16], [96]). In fact, Stanley [8] studied teeth from fossil rodent species and found that body size distributions became progressively skewed towards large size as time went on, whereas the modal size category remained constant; near the small end of the observed range. He suggested that although most of the rodents he studied remained small, a few became larger and were able to invade different niches. Stanley cautioned that because there are smaller than large-bodied mammals, there may be a passive tendency for evolution from small to large body size (taken from Monroe & Bokma [29]).

However, recently Raia *et al.* [97] demonstrated that derived fossil mammal species were characteristically larger, less abundant, had smaller geographic ranges, and persisted for shorter time periods than their smaller ancestors. As these traits are typical of specialist species, these authors proposed that Cope's rule could be explained in terms of the increase in specialization and niche expansion at the clade level, which Cope termed “the law of the unspecialized" [98]. The consequence of this mode of evolution is that narrow ecological specialization results in fewer opportunities for speciation, and thus lower rates of diversification compared to less specialized clades. The extant species of the oryzomyini tribe are characterized by their high level of specialization, possibly derived from generalist ancestors [47]. Over time, larger species have experienced a higher rate of speciation, which agrees with the predictions of Cope's rule and the law of the unspecialized. It is possible that the success of the specialized species and the larger body sizes of the group are a consequence of their wide geographical ranges, which are more susceptible to allopatric speciation events, and thus a higher rate of speciation.

We find no evidence for miniaturization from extant oryzomyine, in accordance with Monroe & Bokma [29] who did not observe any trend for mammals in general. However, our results support Cope's rule in extant species, in accordance with the work of FitzJohn [25], which focused on primates, but determined a different drift model (*i.e.*, a modal curve). These exclusive studies that support Cope's rule using extant taxa suggest that this pattern of evolution is both taxonomic level and model dependent. With respect to taxonomic level, the use of the most inclusive level of mammal diversity could be mixing different trends and histories of body size evolution, and thus obscure the potential trends that can be found in more exclusive monophyletic groups, like Primates and oryzomines. Moreover, recent work by Vendetti *et al.* [99], using the same phylogeny used by Monroe & Bokma [29], demonstrated that different clades of mammals have different rates of body size evolution, and consequently different histories. With respect to model dependence, we suggest that the appropriate way to test trends in body size evolution in extant taxa is to evaluate the fits of different models of body size evolution for particular monophyletic taxa, as we have done here and as has been done by FitzJohn [25].

This evolutionary scenario based on the results of a likelihood-based method of the birth-death model, uses a sample of trees which takes into account phylogenetic uncertainty, and is consistent with the results based on the single phylogenetic tree approach. Both approaches support the tendency for body sizes to increase over time, or Cope's rule (Tables 3, 4, 5, and 6). These similar results are a consequence of the low uncertainty in the phylogenetic history of Oryzomyini tribe, reflected in the higher values of posterior probability in the Bayesian consensus tree (Fig. 1).

In the oryzomyine tribe the evolution of body size began with a small body sized ancestor, which could have colonized the current main distribution in South America from the Northern Hemisphere. Previously, it has been proposed that, given the estimated time for initial diversification of oryzomyines based on molecular studies around 7 Mya [63], [64], the oryzomyine ancestor likely arrived in South America prior to the formation of the Panamanian land bridge at 3.5–4.0 Mya [99], [100] by over-water dispersal. In fact, the ability of oryzomyines to undertake long-distance water dispersal is well-known [101].

We propose that during the early diversification of the tribe, body size increased (*i.e.*, Drift Linear model with a positive trend). The colonization and use of a new and heterogeneous environment, with a wide variability of habitats in South America, allowed for an explosive radiation of Oryzomiyini during the Late Miocene [Fig. 1]. The body size of the Oryzomyini should have undergone change highly influenced by this complex ecological scenario, as observed in the current conspicuous anatomical and ecological deviations from the original generalized bauplan [47], including arboreal, fossorial, dietary and extreme environments specialization [47], [73]. We suggest that during evolutionary history, the consequence of increased body size was an increase in the range of distribution, ultimately increasing the probability of speciation by posterior vicariant processes. The Drift Linear model of evolution supports this idea, given that speciation rate is a linear function of a general trend of body size evolution (Tables 3, 4, 5, and 6). This proposed scenario is coherent with the idea of explosive radiation [64] and Cope's rule, which is observed in the oryzomyine tribe. However, the explosive radiation hypothesis requires an explicit test, utilizing new methods, as proposed by Stadler [102] and Silvestro *et al.* [103] who evaluates the variation of diversification rate over time. Finally, our finding of an increase in body size allows us to reinforce the idea that, in spite of the difficulty of encountering fossils that represent important evolutionary steps, phylogenetic studies of extant taxa can shed new light on the evolutionary history of sigmodontines [60]–[62]. In particular, inter-specific phylogenetic studies are very valuable for describing the origin and radiation of some important traits (like body size). Easy access to these new tools for evaluating evolutionary patterns (*i.e.*, Cope's rule and miniaturization hypotheses) makes it possible to build more complete scenarios using available evidence from extant taxa to complement (or in the absence of) information from the fossil record.

## Supporting Information

Table S1 **Maximum Head-Body length (mm), and GenBank Access Number for Oryzomyini' species used in phylogenetic comparative analysis.** Missing data for maximum head-body length are indicated by gap (−). (*) species used as outgroup.
(DOC)

Nexus Trees S1 **Bayesian tree sample, Bayesian consensus tree, and Maximum Likelihood tree used for comparative analyses.** All trees are calibrated with fossil data (see methods).
(TXT)
