# Correction: Body Size Evolution in Extant Oryzomyini Rodents: Cope's Rule or Miniaturization?

**DOI:** 10.1371/annotation/56843298-87aa-418c-9f6c-db469d0abd20

**Published:** 2012-09-18

**Authors:** Jorge Avaria-Llautureo, Cristián E. Hernández, Dusan Boric-Bargetto, Cristian B. Canales-Aguirre, Bryan Morales-Pallero, Enrique Rodríguez-Serrano

There was an absent parameter (i.e. slope) in the Results section that produces changes in the Results and Discussion. The revised sections are:

Results (second paragraph)

"... where smaller species have higher speciation rates. Similarly, analyses based on a single phylogenetic tree approach and AIC values (Table 5, 6), showed that the same Drift Linear model with a positive trend was the best predictor of body size evolution (AIC = 135.1 and 140.5 for Bayesian consensus and Maximum likelihood trees, respectively). The positive trend for body size evolution was described by θ = 0.48, and θ = 0.45 for Bayesian consensus and Maximum likelihood trees, respectively (Table 5, 6). These results support the general tendency to increase body size over time, and that smaller species have a higher speciation rate (slope = -0.6 and -0.7 for Bayesian consensus and Maximum likelihood trees, respectively)."

Discussion (end of the second paragraph)

"Over time, smaller species have experienced a higher rate of speciation, which agrees with the predictions of Cope's rule and the law of the unspecialized. It is possible that the success of the unspecialized species and the smaller body sizes of the group are a consequence of further dividing the space, making them more susceptible to the effects of genetic drift, and thus a higher rate of speciation."

Discussion (sixth paragraph)

"... ultimately increasing dispersal ability and decreasing the opportunity for speciation. The Drift Linear model of evolution supports this idea, given that speciation rate is a negative linear function of body size evolving with a general positive trend (Tables 3, 4, 5, and..." 

