# Supplementary material for: Body Size Evolution in Extant Oryzomyini Rodents: Cope's Rule or Miniaturization?
Source: PLoS One. 2012 Apr 3;7(4):e34654. doi: 10.1371/journal.pone.0034654 (PMC3318010; doi:10.1371/journal.pone.0034654)
Supplement: Table S1 — Maximum Head-Body length (mm), and GenBank Access Number for Oryzomyini' species used in phylogenetic comparative analysis. Missing data for maximum head-body length are indicated by gap (−). (*) species used as outgroup. (DOC) [file pone.0034654.s001.doc]

|  |  | **GenBank Access Number** | |  |
| --- | --- | --- | --- | --- |
| **Species** | **Max Head-Body length** | **IRBP** | **Cyt-B** | **References** |
| *Aegialomys xanthaeolus* | 133 | GQ178247 | EU074632 | 1, 13 |
| *Amphinectomys savamis* | - | AY163579 | EU579480 | 14 |
| *Cerradomys subflavus* | 179 | AY163626 | AF181274 | 2, 14 |
| *Drymoreomys albimaculatus* | 143 | GU126515 | GU126516 | 7 |
| *Eremoryzomys polius* | 164 | AY163624 | EU579483 | 1, 14 |
| *Euryoryzomys lamia* | 161 | AY163619 | AF181273 | 3, 14 |
| *Euryoryzomys macconnelli* | 172 | AY163620 | U58379 | 4, 14 |
| *Euryoryzomys nitidus* | 163 | EU649041 | U58383 | 4 |
| *Euryoryzomys russatus* | 160 | AY163625 | EF455023 | 4, 14 |
| *Handleyomys alfaroi* | 122 | AY163615 | DQ224409 | 1, 14 |
| *Handleyomys intectus* | 114 | AY163584 | EU579490 | 5, 14 |
| *Holochilus brasiliensis* | 211 | EU273418 | AY041192 | - |
| *Holochilus chacarius* | 195 | AY163586 | DQ227455 | 3,14 |
| *Hylaeamys megacephalus* | 155 | AY163621 | U03538 | 4, 14 |
| *Hylaeamys yunganus* | 149 | AY163629 | DQ224408 | 4, 14 |
| *Melanomys caliginosus* | 135 | AY163590 | EU340020 | 6, 14 |
| *Microryzomys minutus* | 86 | AY163592 | AF108698 | 8, 14 |
| *Neacomys minutus* | 79 | AY163595 | GU126519 | 10, 14 |
| *Neacomys musseri* | 73 | AY163596 | EU579503 | 10, 14 |
| *Neacomys spinosus* | 102 | AY163597 | AF108701 | 11, 14 |
| *Nectomys squamipes* | 245 | AY163598 | EU340012 | 3, 14 |
| *Nephelomys albigularis* | 174 | AY163614 | DQ224407 | 1, 14 |
| *Nesoryzomys narboroughi* |  | AY163600 | GU126523 | 7, 14 |
| *Nesoryzomys swarthi* | - | AY163601 | EU340014 | 14 |
| *Oecomys bicolor* | 118 | AY163604 | AJ496307 | 9, 14 |
| *Oecomys catherinae* | 150 | AY163605 | EU579507 | 3, 14 |
| *Oecomys concolor* | 149 | AY163606 | FJ361052 | 12, 14 |
| *Oecomys mamorae* | 160 | AY163607 | EU579509 | 3, 14 |
| *Oecomys superans* | 176 | AY277464 | AY275123 | 3, 15 |
| *Oecomys trinitatis* | 140 | AY163608 | U58390 | 10, 14 |
| *Oligoryzomys chacoensis* | 130 | EU649059 | AY275706 | 3, 14 |
| *Oligoryzomys flavescens* | 113 | AY163609 | DQ826012 | 3, 14 |
| *Oligoryzomys fornesi* | 84 | AY163610 | AY452199 | 3, 14 |
| *Oligoryzomys fulvescens* | 99 | AY163611 | EU294249 | 3, 14 |
| *Oligoryzomys messorius* |  | DQ826032 | DQ826024 | 16 |
| *Oligoryzomys moojenis* | 96 | DQ826031 | DQ826017 | 3, 16 |
| *Oligoryzomys nigripes* | 120 | AY163612 | EU258551 | 3, 14 |
| *Oligoryzomys stramineus* | 111 | AY163613 | DQ826027 | 3, 14 |
| *Oreoryzomys balneator* | 100 | AY163617 | EU579510 | 1, 14 |
| *Oryzomys couesi* | 142 | AY163618 | EU074665 | 6, 14 |
| *Handleyomys melanotis* |  | AY163622 | GU126541 | 14 |
| *Oryzomys mexicanus* |  | GQ178252 | GQ178246 | 13 |
| *Oryzomys palustris* | 150 | AY163623 | EU074639 | 1, 14 |
| *Pseudoryzomys simplex* | 127 | AY163633 | EF621306 | 3, 14 |
| *Scolomys juruaense* |  | AY277478 | AF108696 | 15, 17 |
| *Scolomys ucayalensis* | 163 | AY163638 | AF527421 | 3, 14 |
| *Sigmodontomys alfari* | - | AY163641 | EU340016 | 14 |
| *Sooretamys angouya* | 185 | EU649072 | EF455034 | 3 |
| *Transandinomys talamancae* | 142 | AY163627 | EU579515 | 4, 14 |
| *Zygodontomys brevicauda* | 140 | AY163645 | EU579521 | 3, 14 |
| *Zygodontomys cherriei* |  | AY163646 | GU126550 | 7, 14 |
| *Bibimys labiosus ** | - | AY277436 | DQ444329 | 15 |
| *Juliomys pictipes** | - | AY277451 | EF127514 | 15, 18 |
| *Calomys callosus ** | - | AY277440 | DQ447282 | 15 |
| *Eligmodontia typus ** | - | AY277445 | EU377643 | 15,19 |

**References**

1. Weksler M (2006) Phylogenetic relationships of oryzomyine rodents (Muroidea: Sigmodontinae): separate and combined analyses of morphological and molecular data. Bull Amer Mus Nat Hist 196: 1-149.

2. Percequillo AR, Hingst-Zaher E, Bonvicino CR (2008) Systematic Review of Genus *Cerradomys* Weksler, Percequillo and Voss, 2006 (Rodentia: Cricetidae: Sigmodontinae: Oryzomyini), with Description of Two New Species from Eastern Brazil. Am Mus Novit 3622: 1-46.

3. Bonvicino CR, Oliveira JA, D'Andrea PS (2008) Guía dos Roedores do Brasil, com chaves para gêneros baseadas em caracteres externos. Rio de Janeiro: Centro Pan-Americano de Febre Aftosa – OPAS/OMS. 120 pp.

4. Musser GG, Carleton MD, Brothers E, Gardner AL (1998) Systematic studies of Oryzomyine rodents (Muridae, Sigmodontinae): diagnoses and distributions of species formerly assigned to Oryzomys ‘‘capito’’. Bull Amer Mus Nat Hist 236: 1-376.

5. Voss RS, Gómez-Laverde M, Pacheco V (2002) A New Genus for *Aepeomys fuscatus* Allen, 1912, and *Oryzomys intectus* Thomas, 1921: Enigmatic Murid Rodents from Andean Cloud Forests. Am Mus Novit 3373: 1-42.

6. Reid FA (1997) A Field Guide to the Mammals of Central America and Southeast Mexico. New York: Oxford University Press. 346 p.

7. Percequillo AR, Weksler M, Costa LP (2011) A new genus and species of rodent from the Brazilian Atlantic Forest (Rodentia: Cricetidae: Sigmodontinae), with comments on the Oryzomyine biogeography. Zool J Linn Soc 161:357-390

8. Anderson S (1997) Mammals of Bolivia, taxonomy and distribution. Bull Amer Mus Nat Hist 231: 1-651.

9. Voss RS, Simmons N, Lunde G (2001) The Mammals of Paracou, French Guiana: a Neotropical lowland rainforest fauna, Part 2: Nonvolant species. Bull Amer Mus Nat Hist 263: 1-236.

10. Patton JL, Da Silva MNF, Malcolm JR (2000) Mammals of the Rio Juruá and the evolutionary and ecological diversification of Amazonia. Bull Amer Mus Nat Hist 244: 1-306.

11. Luna L, Patterson BD (2003) A Remarkable New Mouse (Muridae: Sigmodontinae) from Southeastern Peru: With Comments on the Affinities of *Rhagomys rufescens* (Thomas, 1886). Fieldiana Zool, N.S. 101: 1–24.

12. Redford KH, Eisenberg JF (1992) Mammals of the Neotropics, Volume 2: The Southern Cone: Chile, Argentina, Uruguay, Paraguay. University of Chicago Press. 430 p.

13. Hanson JD, Indorf JL, Swier VJ, Bradley RD (2010) Molecular divergence within the *Oryzomys palustris* complex: evidence for multiple species. J Mammal 91(2):336-347.

14. Weksler M (2003) Phylogeny of Neotropical oryzomyine rodents (Muridae: Sigmodontinae) based on the nuclear IRBP exon. Mol Phyl Evol 29: 331-349

15. D'Elia G (2003) Phylogenetics of Sigmodontinae (Rodentia, Muroidea, Cricetidae), with special reference to the akodont group, and with additional comments on historical biogeography. Cladistics 19:307-323.

16. Miranda GB, Oliveira LF, Andrades-Miranda J, Langguth A, Callegari-Jacques SM, Mattevi MS (2009) Phylogenetic and phylogeographic patterns in sigmodontine rodents of the genus oligoryzomys. J Hered 100:309-321.

17. Smith MF, Patton JL (1999) Phylogenetic relationships and the radiation of sigmodontine rodents in South America: evidence from cytochrome b. J Mammal Evol 6: 89-128.

18. Costa LP, Pavan SE, Leite YLR, Fagundes V (2007) A new species of Juliomys (Mammalia: Rodentia: Cricetidae) from the Atlantic forest of southeastern Brazil. Zootaxa 1463: 21-37

19. Mares MA, Braun JK, Coyner BS, Van Den Bussche RA (2008) Phylogenetic and biogeographic relationships of gerbil mice Eligmodontia (Rodentia, Cricetidae) in South America, with a description of a new species. Zootaxa 1753: 1-33
